# Molecule Set Comparator (MSC): a CDK-based open rich‐client tool for molecule set similarity evaluations

**DOI:** 10.1186/s13321-021-00485-4

**Published:** 2021-02-01

**Authors:** Kohulan Rajan, Jan-Mathis Hein, Christoph Steinbeck, Achim Zielesny

**Affiliations:** 1 Institute for Inorganic and Analytical Chemistry, Friedrich-Schiller-University Jena, Lessingstr. 8, Jena, 07745 Germany; 2 Institute for Bioinformatics and Chemoinformatics, Westphalian University of Applied Sciences, August-Schmidt-Ring 10, 45665 Recklinghausen, Germany

**Keywords:** Molecule set comparison, Chemistry Development Kit, CDK, Descriptor, Machine learning

## Abstract

The open rich-client Molecule Set Comparator (MSC) application enables a versatile and fast comparison of large molecule sets with a unique inter-set molecule-to-molecule mapping obtained e.g. by molecular-recognition-oriented machine learning approaches. The molecule-to-molecule comparison is based on chemical descriptors obtained with the Chemistry Development Kit (CDK), such as Tanimoto similarities, atom/bond/ring counts or physicochemical properties like logP. The results are summarized and presented graphically by interactive histogram charts that can be examined in detail and exported in publication quality.
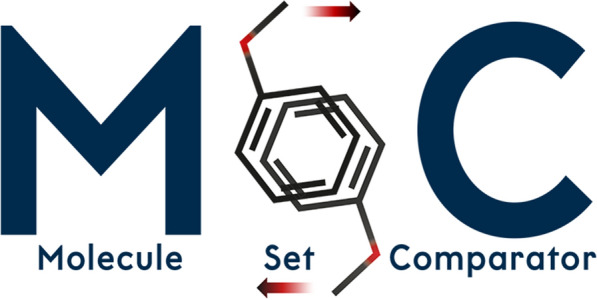

The comparison of molecules lies at the heart of cheminformatics from its beginnings with molecular comparative studies addressing a wide range of research activities [1]. A variety of molecular comparisons may be computationally performed with open cheminformatics libraries like RDKit [2], Indigo [3] or CDK [4–8] driven by appropriate scripting solutions (which require programming skills) or with open rich-client applications like DataWarrior [9, 10] or Scaffold Hunter [11–13] (which are accessible to scientific end-users). Halfway between scripting solutions and rich-clients there are pipelining-workflow systems like the open analytics platform KNIME [14, 15] that offer specific worker nodes—which themselves may be based on open cheminformatics libraries like the RDKit [16], Indigo [17] or CDK [18] nodes for KNIME—that can be flexibly connected to construct automated molecule comparison workflows where the node composition is comfortably supported by a graphical editor.

Besides the frequent use cases, which are already covered by available solutions, current machine learning tasks make demands on dedicated molecule-to-molecule comparisons, which have to be addressed by new specific applications to effectively support corresponding research activities.

“Intelligent” molecular recognition systems based on new deep learning approaches in cheminformatics try to predict a molecule in question (the system’s output) from a specific molecular representation (the system’s input), where the input representation of the desired molecule may be a set of its molecular features, a pixel image of the molecule’s 2D structure or any other encoding that relates to the original molecule. To assess the predictive power of a molecular recognition system, the predicted molecules have to be comprehensively compared with their corresponding original molecules that were used to create the molecular representation for the system’s input. For these comparisons a fingerprint based Tanimoto similarity between original and predicted molecule may be used or the difference of their atom/ring counts may be calculated. Also differences regarding their physico-chemical properties like logP may be of interest. For large sets of original and corresponding predicted molecules these similarity or difference values may be neatly summarized by frequency histograms which then allow for a versatile and fast assessment of the recognition abilities of the machine with regard to the selected comparator. The new Molecule Set Comparator (MSC) application focuses on these comparisons and aims to alleviate them.

MSC is a Java rich-client end-user application which architecturally follows a Model-View-Controller (MVC) pattern [19] and utilizes JavaFX [20] for graphical user interface (GUI) design and charting. All molecular operations are performed with the Chemistry Development Kit (CDK) [4–8]. Graphical image generation is realized with the PDFBox library [21] and the Batik SVG toolkit [22].

Figure 1 shows the MSC input view with molecule sets and comparative chemical descriptor selection. Supported molecule set formats are SMILES or SDF text files. The first set of molecules (e.g. a text file with a single SMILES string in each line) should contain the original molecules from which specific molecular representations have been derived to be used as input for the molecular recognition system. The second molecule set should contain the molecules predicted by the molecular recognition system at a corresponding position (i.e. the SMILES string of the predicted molecule must be on the same line as its original molecule in the first set of molecules). It should be noted that the order of the two molecule sets to be specified does not affect the subsequent comparative evaluations since these rely on absolute molecule-pair properties only, i.e. the molecule sets could be mutually interchanged without any effect.

**Fig. 1:**
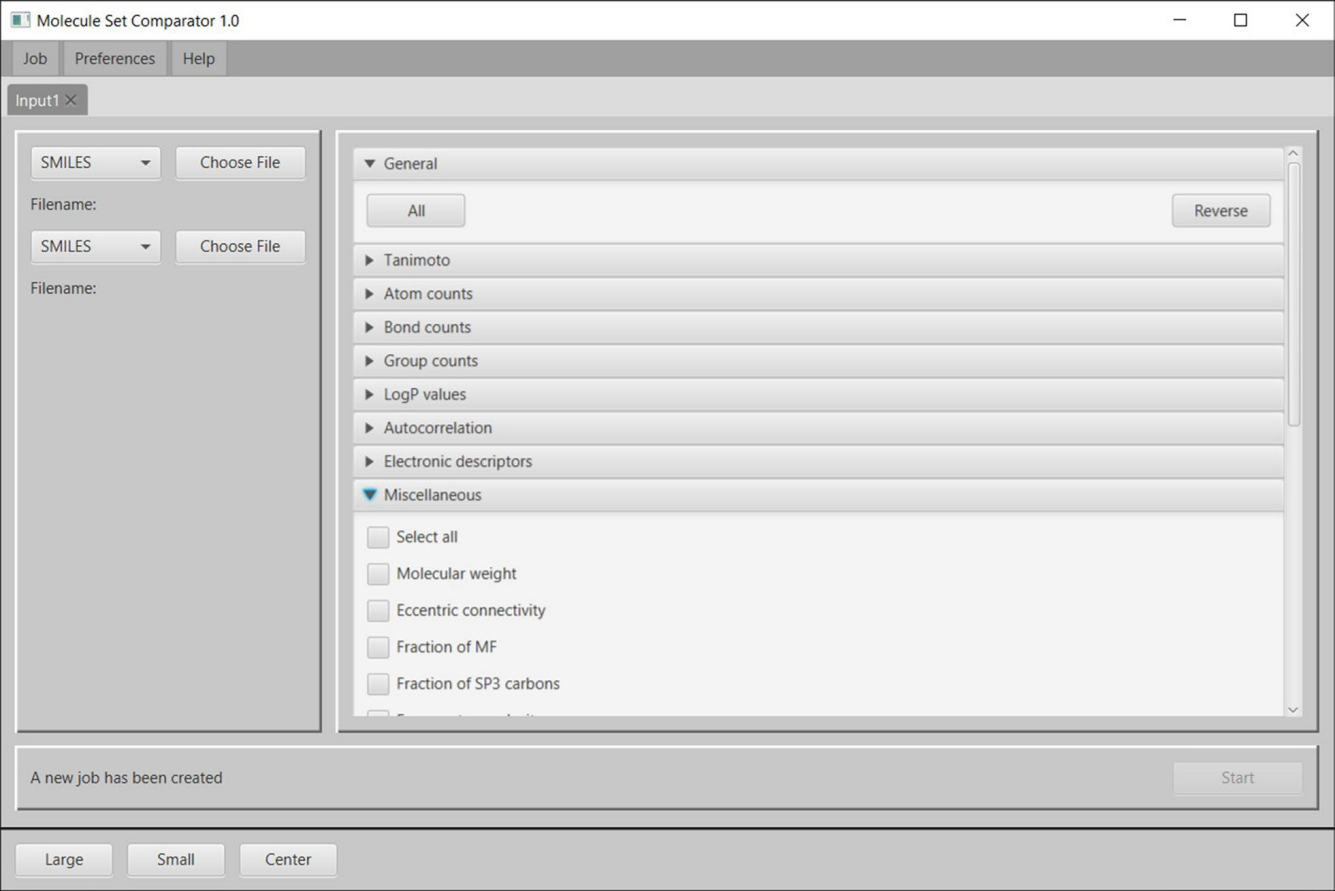
MSC input view with molecule sets selection and comparator choice for subsequent molecule comparisons

The available descriptors for original/predicted molecule comparison are summarized in Table 1. The Tanimoto similarity directly refers to an original/predicted molecule pair, for all other numerical descriptors the absolute difference between the descriptor value of the original and the predicted molecule is calculated. The resulting Tanimoto similarities and absolute descriptor value differences of the original/predicted molecule pairs are then used for the particular histogram binnings in the following.

**Table 1:** Available molecular descriptors for original/predicted molecule comparison

Descriptor type	Available descriptors in MSC
Tanimoto similarity	Basic, LINGO, Extended, E-State, PubChem, Shortest Path, Substructure
Atom counts	All atoms, Carbon, Oxygen, Sulphur, Nitrogen, Phosphor, Aromatic atoms, Spiro atoms, C1SP1, C2SP1, C1SP2, C2SP2, C3SP2, C1SP3, C2SP3, C3SP3, C4SP3
Bond counts	All bonds, Aromatic bonds, Single, Double, Triple, Quadruple, Rotatable
Group counts	Acidic groups, Basic groups, All small rings, Aromatic rings, Rings of size 3–9, All rings, H-bond acceptors, H-Bond donors
LogP values	Mannhold LogP, JPLogP, XLogP, ALogP, ALogP2
Autocorrelation	ATS charge, ATS mass, ATS polarizability
Electronic descriptors	Atomic polarizability, Bond polarizability, Fractional PSA, Topological PSA, Molar refractivity
Miscellaneous	Molecular weight, Eccentric connectivity, FMF, SP3 fraction, Fragment complexity, 1–3. kappa shape index, Largest pi system, Largest chain, Longest aliphatic chain, Petitjean number, Petitjean shape index, VdW volume, Vertex adjacency, Weighted path descriptor, Wiener path number, Wiener polarity number, Zagreb index, Equality

The MSC input view allows the concurrent selection of multiple descriptors for original/predicted molecule comparisons. A comparative histogram chart is then generated for each selected descriptor (see Fig. 2): each histogram consists of a number of bars where each bar comprises a specific range of evaluated similarity or absolute descriptor difference values: The height of a bar corresponds to the number of molecule pairs whose similarity/absolute descriptor difference value lies within the bar’s value range. The default number of bars is 10 and the default value for the lower border of the first bar and the upper border of the last bar are set to the minimal and maximal similarity/absolute descriptor difference values respectively.

Figure 2 depicts the calculated output view for a JPlogP-descriptor-based comparison as an example: The numerical differences between the JPlogP values of all original/predicted molecule pairs are evaluated and binned according to their absolute difference values in order to match the specified number of bars of the histogram. The highest left bar contains 56.9 % of all original/predicted molecule pairs which have an absolute JPlogP difference value between 0 and 0.5, the second next left bar contains 22.5 % of all molecule pairs with an absolute JPlogP difference between 0.5 and 1 etc. (note, that the bin labels describe the upper bin border value in this case). For documentation purposes, the *Summarize* button (see Fig. 2 top left) can be used to retrieve a textual summary containing the calculated results together with some of their statistical characteristics (mean, minimum and maximum value). Each histogram chart can be comfortably configured with sliders for lower/upper bar borders or an input field for definition of the desired number of bars. In addition, bar borders may be arbitrarily adjusted via a separate dialog (see Fig. 3). Bar labels or the y-axis range may also be changed on-the-fly and bars may be labelled with their frequencies. Charts can be exported in arbitrarily high quality to different graphics formats (PNG, JPEG, PDF, or SVG). For an interactive exploration of the original/predicted molecule pairs behind a specific bar, this bar may be clicked to open a modal window which allows for navigation through all the corresponding original/predicted molecule pairs that sum up to the bar’s height/frequency: For the displayed molecule pair in Fig. 2 the comparison result, (i.e. the calculated absolute JPlogP difference value) is 0.48891 where additional descriptors like *Basic* or *PubChem fingerprint* may be selected and calculated on-the-fly to inspect further similarities and differences. The molecular images may also be saved as PNG, JPEG, PDF, or SVG files. A calculated output view can be permanently saved and reloaded for later use.

**Fig. 2:**
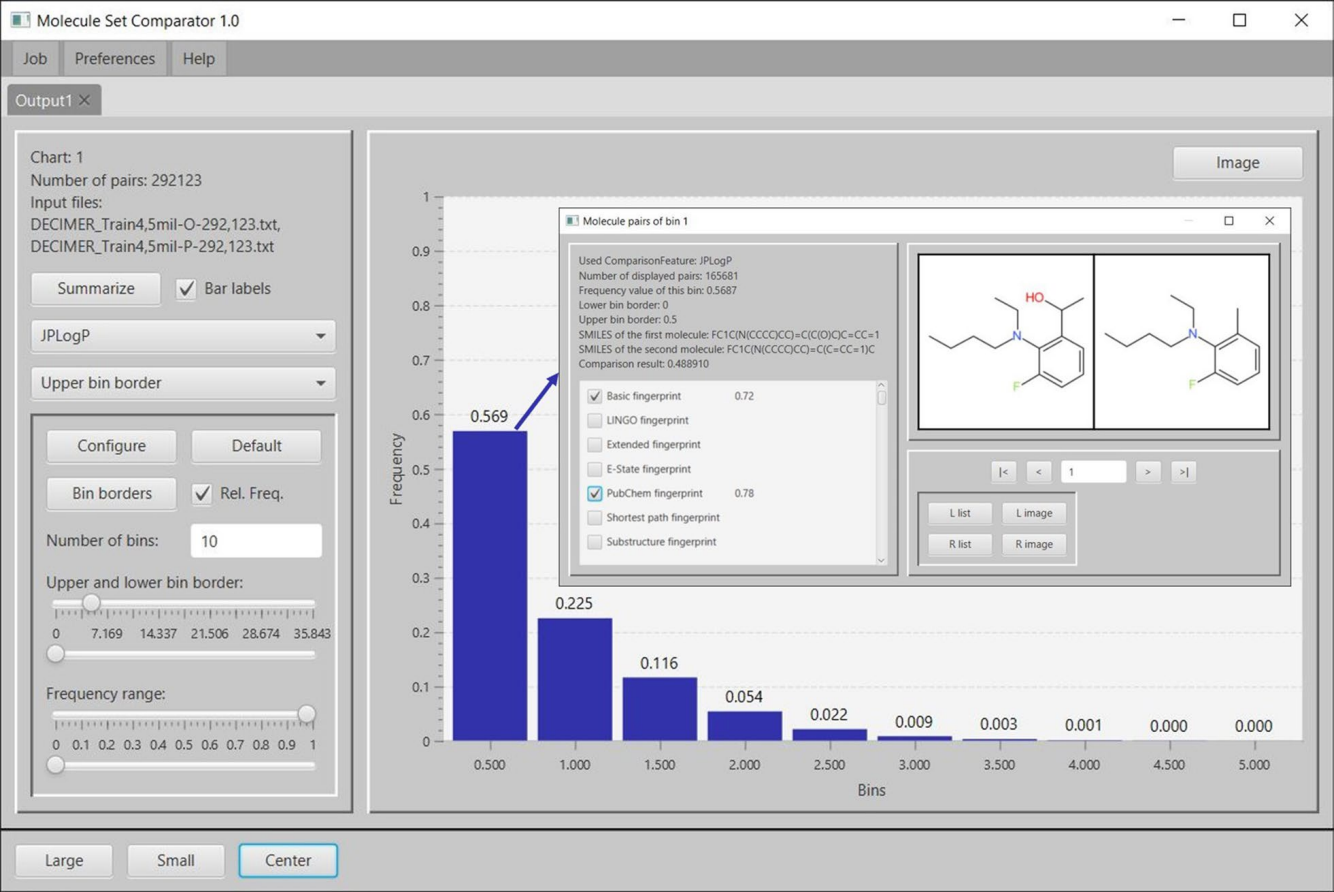
Output view with an interactive comparative histogram chart. A histogram-bar related modal window (see arrow) provides detailed information about the corresponding original/predicted molecule pairs

**Fig. 3:**
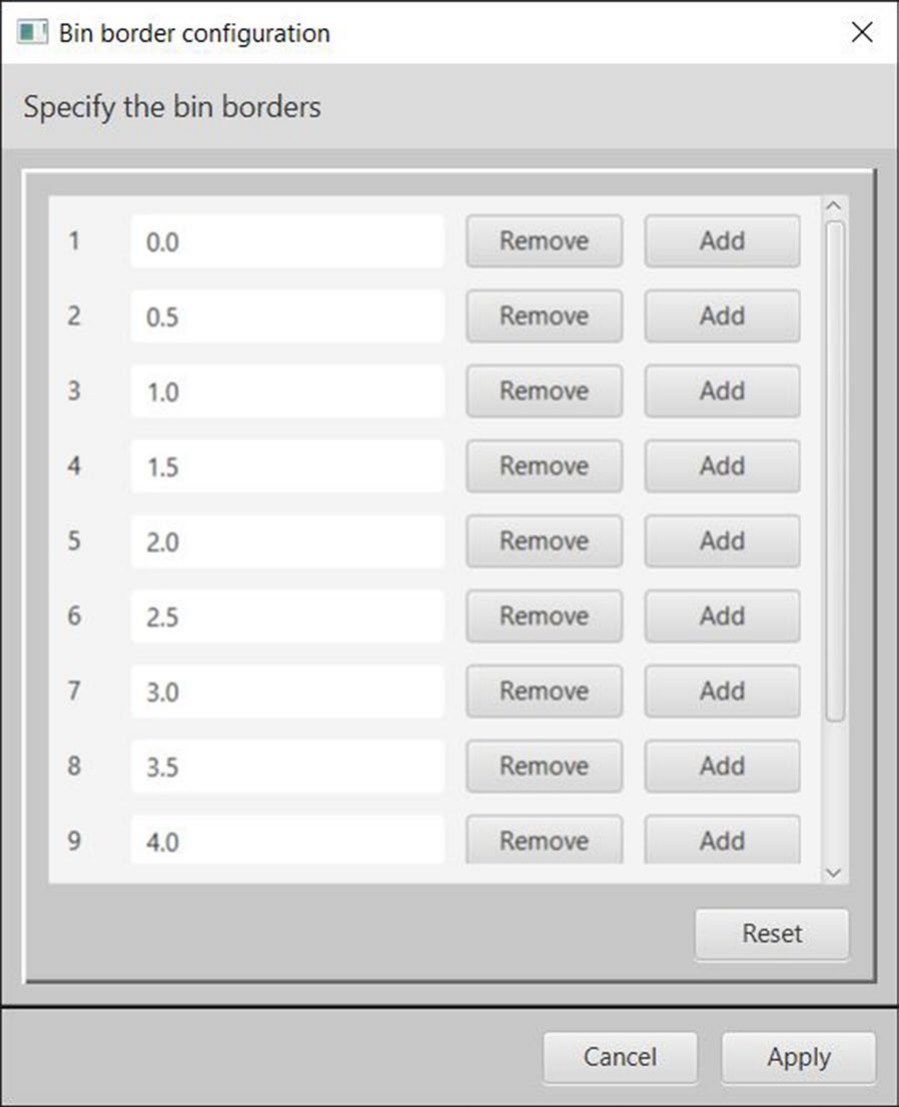
Configuration dialog for arbitrary bar border definitions

MSC offers several preference settings: Default output directories for images, molecule lists, summary reports, calculated results, or a default input directory for molecule sets. Also, the number of parallel threads for comparative molecule calculations, the number of molecule pairs for MSC output, the default number of histogram bins, or the image quality can be specified. Preferences are permanently saved as an XML file in the *MSC_Files* directory of the MSC start directory.

MSC supports concurrent calculations via the *Parallel threads* preference which may considerably reduce computing times. For up to eight concurrent calculation threads MSC performs nearly inversely proportional to the number of threads (acceleration factor of 7.4 for an Intel Xeon Gold 6254 18-core processor, on a workstation running with Windows 10 Pro for Workstations, using 256 gigabytes of RAM). Using more than 8 threads still yields improvement up to 16 threads (factor of 11.9 on the same machine). On average, a (basic CDK fingerprint) Tanimoto similarity comparison of one million original/predicted molecule pairs using 8 calculation threads and 16 gigabytes of RAM takes less than 3 min.

The MSC GitHub repository contains the complete source code, all used libraries, installation instructions for all major platforms, a Gradle project for Netbeans as well as supplementary tutorials for installation, overview and application.

## Conclusions

MSC is a versatile and fast end-user tool for comparing large molecule sets containing millions of chemical structures. As a rich client it does not require any programming skills and runs on all major platforms (Windows, Linux, and MacOS). A major application area is the support of molecular-recognition oriented machine learning tasks that require a large-scale and thorough comparative analysis of molecular features: The MSC tool allows to replace tedious scripting approaches with cumbersome manual PDF views by fast, flexible and comprehensive graphical point-and-click inspections. In addition to saving time, the new open tool may provide insights that might have been overlooked otherwise.

## Availability and requirements

Project name: MSC.

 Project home page: MSC repository at https://github.com/zielesny/MSC.

Operating system(s): Platform independent.

Programming language: Java.

Other requirements: JavaFX 14 [20], CDK 2.3 [4], PDFBox 2.0.17 [21], Batik SVG Toolkit 1.13 [22], Apache Commons Logging 1.2 [23].

License: GNU General Public License version 3.

## Data Availability

MSC repository at https://github.com/zielesny/MSC.

## References

[1] Gasteiger J, Engel T (2018). Chemoinformatics. Basic concepts and methods.

[2] RDKit: Open-source cheminformatics software. http://rdkit.org/. Accessed 10 Sept 2020.

[3] Indigo Toolkit. https://lifescience.opensource.epam.com/indigo/. Accessed 17 Dec 2020.

[4] Chemistry Development Kit (CDK). https://cdk.github.io/. Accessed 01 June 2020

[5] Willighagen EL, Mayfield JW, Alvarsson J, Berg A, Carlsson L, Jeliazkova N, Kuhn S, Pluskal T, Rojas-Chertó M, Spjuth O, Torrance G, Evelo CT, Guha R, Steinbeck C (2017). The Chemistry Development Kit (CDK) v2.0: atom typing, depiction, molecular formulas, and substructure searching. J Cheminform..

[6] May JW, Steinbeck C (2014). Efficient ring perception for the Chemistry Development Kit. J Cheminform..

[7] Steinbeck C, Hoppe C, Kuhn S, Floris M, Guha R, Willighagen EL (2006). Recent Developments of the Chemistry Development Kit (CDK)—an open-source java library for chemo- and bioinformatics. Curr Pharm Des.

[8] Steinbeck C, Han Y, Kuhn S, Horlacher O, Luttmann E, Willighagen EL (2003). The Chemistry Development Kit (CDK): An Open-Source Java Library for Chemo- and Bioinformatics. J Chem Inf Comput Sci.

[9] DataWarrior. http://openmolecules.org/datawarrior/. Accessed 10 Sept 2020.

[10] Sander T, Freyss J, von Korff M, Rufener C (2015). DataWarrior: an open-source program for chemistry aware data visualization and analysis. J Chem Inf Model.

[11] Wetzel S, Klein K, Renner S, Rauh D, Oprea TI, Mutzel P, Waldmann H (2009). Interactive exploration of chemical space with Scaffold Hunter. Nat Chem Biol.

[12] Schäfer T, Kriege N, Humbeck L, Klein K, Koch O, Mutzel P (2017). Scaffold Hunter: a comprehensive visual analytics framework for drug discovery. J Cheminform.

[13] Scaffold Hunter. http://scaffoldhunter.sourceforge.net/. Accessed 17 Dec 2020.

[14] Berthold MR, Cebron N, Dill F, Gabriel TR, Koetter T, Meinl T, Ohl P, Sieb C, Thiel K, Wiswedel B, Preisach C, Burkhardt H, Schmidt-Thieme L, Decker R (2008). KNIME: the konstanz information miner. Data analysis, machine learning and applications. studies in classification, data analysis, and knowledge organization.

[15] KNIME Analytics Platform. https://www.knime.com/knime-analytics-platform. Accessed 17 Dec 2020.

[16] RDKit Nodes for KNIME. https://www.knime.com/rdkit. Accessed 17 Dec 2020.

[17] Indigo Nodes for KNIME. https://www.knime.com/community/indigo. Accessed 17 Dec 2020.

[18] CDK Nodes for KNIME. https://www.knime.com/community/cdk. Accessed 17 Dec 2020.

[19] Reenskaug TMH, Xerox MVC PARC (1978-79) http://heim.ifi.uio.no/~trygver/themes/mvc/mvc-index.html. Accessed 01 June 2020.

[20] JavaFX. https://openjfx.io/. Accessed 01 June 2020.

[21] Apache PDFBox | A Java PDF Library. https://pdfbox.apache.org/. Accessed 01 June 2020.

[22] Apache Batik—Apache XML Graphics—Apache Software. https://xmlgraphics.apache.org/batik/. Accessed 01 June 2020.

[23] Apache Commons Logging—Overview. https://commons.apache.org/proper/commons-logging/. Accessed 01 June 2020.

